# Inter-pregnancy interval and risk of recurrent pre-eclampsia: systematic review and meta-analysis

**DOI:** 10.1186/s12978-016-0197-x

**Published:** 2016-07-18

**Authors:** Gabriela Cormick, Ana Pilar Betrán, Agustín Ciapponi, David R. Hall, G. Justus Hofmeyr, on behalf of the calcium and Pre-eclampsia Study Group

**Affiliations:** Department of Mother and Child Health Research, Institute for Clinical Effectiveness and Health Policy (IECS), Emilio Ravignani 2024, Buenos Aires, Argentina; Centro de Investigaciones en Epidemiología y Salud Pública (CIESP, CONICET-IECS), Buenos Aires, Argentina; Department of Reproductive Health and Research, World Health Organization, Avenue Appia 20, Geneva, 1211 Switzerland; Argentine Cochrane Branch, Institute for Clinical Effectiveness and Health Policy (IECS), Emilio Ravignani 2024, Buenos Aires, Argentina; Department of Obstetrics and Gynaecology, Stellenbosch University and Tygerberg Hospital, Cape Town, South Africa; Effective Care Research Unit, Eastern Cape Department of Health, University of the Witwatersrand, University of Fort Hare, Walter Sisulu University, East London, South Africa

**Keywords:** Recurrence, Pre-eclampsia, Eclampsia, Inter-pregnancy interval, Birth interval, Meta-analysis, Systematic review, Birth spacing, Hypertensive disorders of pregnancy

## Abstract

**Background:**

Women with a history of pre-eclampsia have a higher risk of developing pre-eclampsia in subsequent pregnancies. However, the role of the inter-pregnancy interval on this association is unclear.

**Objective:**

To explore the effect of inter-pregnancy interval on the risk of recurrent pre-eclampsia or eclampia.

**Search strategy:**

MEDLINE, EMBASE and LILACS were searched (inception to July 2015).

**Selection criteria:**

Cohort studies assessing the risk of recurrent pre-eclampsia in the immediate subsequent pregnancy according to different birth intervals.

**Data collection and analysis:**

Two reviewers independently performed screening, data extraction, methodological and quality assessment.

Meta-analysis of adjusted odds ratios (aOR) with 95 % confidence intervals (CI) was used to measure the association between various interval lengths and recurrent pre-eclampsia or eclampsia.

**Main results:**

We identified 1769 articles and finally included four studies with a total of 77,561 women. The meta-analysis of two studies showed that compared to inter-pregnancy intervals of 2–4 years, the aOR for recurrent pre-eclampsia was 1.01 [95 % CI 0.95 to 1.07, I^2^ 0 %] with intervals of less than 2 years and 1.10 [95 % CI 1.02 to 1.19, I^2^ 0 %] with intervals longer than 4 years.

**Conclusion:**

Compared to inter-pregnancy intervals of 2 to 4 years, shorter intervals are not associated with an increased risk of recurrent pre-eclampsia but longer intervals appear to increase the risk. The results of this review should be interpreted with caution as included studies are observational and thus subject to possible confounding factors.

**Electronic supplementary material:**

The online version of this article (doi:10.1186/s12978-016-0197-x) contains supplementary material, which is available to authorized users.

## Background

Pre-eclampsia is a gestational disorder usually defined as hypertension accompanied by proteinuria [1]. It remains a major cause of maternal and neonatal mortality and morbidity worldwide [2]. Pre-eclampsia can lead to severe complications such as eclampsia, liver rupture, stroke, renal failure, or eodema in the mother and fetal growth restriction and pre-term birth in the newborn. A recent revised definition proposes proteinuria not to be required for the diagnosis of pre-eclampsia as long as there is a persistant high blood pressure associated with one or more severe complications or adverse conditions [1, 3]. Women with a history of pre-eclampsia have a higher risk of developing pre-eclampsia in subsequent pregnancies [4–6]. This risk of recurrent pre-eclampsia varies from 7 to 65 % depending on factors such as gestational age at the onset or delivery of the initial pregnancy, severity of the disease and women’s pre-existing medical disorders [6].

The relationship between birth interval and maternal and perinatal outcomes has been studied extensively [7, 8]. Short inter-pregnancy intervals (< 18 months) may be associated with adverse pregnancy outcomes due to depletion of maternal nutrients and to the failure to treat existing co-morbidities [9, 10]. Whereas longer inter-pregnancy intervals might allow more complete recovery of the mother, they are associated with reduced fertility, older age, maternal disorders and partner change that are also linked with higher risk of pre-eclampsia [11]. A recent analysis of 894,476 women with consecutive pregnancies in 18 Latin American countries showed that longer birth intervals had increased odds of pre-eclampsia [8].

However, for those women who develop pre-eclampsia, it is not clear whether the inter-pregnancy interval is associated with the risk of recurrent pre-eclampsia in the following pregnancy. A Norwegian study of 19,970 women with pre-eclampsia found that the risk of having recurent pre-eclampsia in the following pregnancy tended to decrease with increasing time interval between deliveries, although the results were not significant [12]. Two other studies found no difference in the recurrence of pre-eclampsia according to inter-pregnancy interval [13, 14].

In 2010 the National Institute for Health Care Excellence (NICE) Clinical guidelines for the management of hypertensive disorders during pregnancy reported that there is no increased risk of recurrent pre-eclampsia with inter-pregnancy intervals up to 10 years, but the level of evidence was derived from a single well-conducted cohort study, with low risk of bias [15]. In their 2014 update, the Society of Obstetricians and Gynaecologists of Canada (SOGC) also remarked that there is no increased risk of pre-eclampsia recurrence with longer inter-pregnancy intervals. However there are some reports stating that there is an increased recurrent risk with interpregnancy intervals of less than 2 years or more than 10 years and there are other or not providing recommendations on this topic due to insufficient evidence [16–18].

We conducted a systematic review to explore the effect of inter-pregnancy interval on the risk of recurrent pre-eclampsia or eclampsia. This information is important to improve shared decision making of health care providers and women who had pre-eclampsia or eclampsia in the previous pregnancy and plan to become pregnant again.

## Methods

We followed the reporting recommendations of the PRISMA statement and the Meta-analysis Of Observational Studies in Epidemiology group (MOOSE) [19, 20]. This review was registered in Prospero Centre for Reviews and Dissemination, University of York: Systematic review on inter-pregnancy interval and risk of recurrent pre-eclampsia: CRD42015016682 [21].

### Criteria for considering studies for this review

The studies included in this review required the following criteria:

- Type of studies: observational prospective or retrospective cohort studies assessing the risk of recurrent pre-eclampsia in the immediate subsequent pregnancy according to different birth interval periods.
- Type of participants: woman with a history of pre-eclampsia or eclampsia in a singleton pregnancy. We included studies with participants of any age, ethnic group, parity, education and socio-economic status fulfilling the previous criteria. Very specific groups of women (e.g. women with systemic lupus, erythematosus lupus, rheumatoid arthritis or diabetes) were not included.
- Types of birth intervals: We included any type of birth interval definition; e.g. *inter*-*pregnancy* interval defined as the time between the date that the first pregnancy ended and the date of the last menstrual period for the second pregnancy; or *inter*-*birth intervals* defined as the interval between the dates of two consecutive births from two separate pregnancies.
- Type of outcomes: our primary outcomes were recurrent pre-eclampsia or eclampsia in the second pregnancy irrespective of the severity, gestational age at onset and definition. Any definition of pre-eclampsia described by the authors was accepted.

In the studies included, the following outcomes were also considered: (1) Incidence of hypertension in the second pregnancy irrespective of severity, time of onset or definition used and (2) Perinatal outcomes in the second pregnancy: fetal and neonatal death, low birth weight defined as weight at birth < 2.5 kg, and preterm birth defined as gestational age < 37 weeks at delivery.

### Search strategy for identification of studies

The search strategy was developed with the assistance of a librarian experienced in electronic search strategies for systematic reviews from the Institute of Clinical Effectiveness (IECS) and tested by a second expert in search strategies from the World Health Organization (WHO). We searched MEDLINE (1966 to Jul 7, 2015), EMBASE (1980 to Jul 7, 2015) and LILACS (1982 to Jul 7, 2015) using a combination of medical subject headings, key word terms and word variants for birth spacing and adverse outcomes. The MEDLINE search strategy was translated into the other databases using the appropriate controlled vocabulary as applicable. This review had no language restrictions. Additional file 1 presents the search strategy developed for this systematic review.

We searched systematic reviews and meta-analyses from these databases to check their reference lists. We checked the reference lists of primary studies selected for full-text evaluation for additional potentially relevant articles not identified by the electronic search. Authors of relevant papers were contacted regarding any further published or unpublished work. Authors of manuscripts reporting incomplete information were contacted to provide the missing information. ISI Web of Science was searched for papers citing studies included in the review.

### Process of study identification, selection and data extraction

Citations identified in the electronic databases were imported in *Early Reviewer Organizing Software* (EROS) and duplicates deleted [22]. EROS is a web-based software designed specifically to perform the first stages of systematic review by organizing the initial phases, distributing the workload, facilitating independent revision of references and resolution of discrepancies, and incorporating quality assessment. Two investigators (GC and APB) independently screened the titles and abstracts to select potentially relevant citations for full text evaluation. Discrepancies were resolved through discussion and consensus. When citations were considered relevant or when information in the title/abstract was insufficient for decision on inclusion/exclusion criteria, the full text was retrieved and evaluated.

A structured data-extraction form specifically designed according to the information needs of this review was created in Excel® to conduct data extraction. Two reviewers (GC and APB) extracted the data from the included citations independently. Data extraction from the two reviewers was compared and discrepancies were discussed until consensus was reached.

The information extracted from each study was the following: Study identification information: author, publication year, country; study characteristics: design, data collection period; type of women included; sample size: number of women with pre-eclampsia in the first pregnancy; number of women who developed pre-eclampsia in the second pregnancy; definitions of pre-eclampsia in the two pregnancies and verification of diagnosis; and definition of birth interval. Crude and adjusted RR or OR, confidence intervals and adjustment factors were also extracted. Information on change of partner, incidence of hypertension and perinatal outcomes in the index pregnancy were collected if data were available. When required, data was extracted from the article’s graphs by using Adobe Acrobat X1 pro. All imputed values and numeric calculations were confirmed by a third person.

### Risk of bias assessment

We assessed data quality in each included study using The Newcastle-Ottawa Scale (NOS) for non-randomised studies in meta-analyses [23]. Two reviewers (GC and APB) independently assessed the quality of each included study, discrepancies were discussed and if consensus was not reached a third reviewer was consulted (AC). The NOS scale for cohort studies assesses three main domains. The first domain evaluates the selection of the exposed, non-exposed cohorts and the ascertainment of exposure. For the purpose of this review, we defined a study as at low risk of bias if the exposed cohort was derived from the general community, if the non-exposed cohort belonged to the same group as the exposed, and if there was documentation that the dates used to calculate the inter-birth interval were extracted from clinical records. On the other hand the study was classified as at high risk of bias if the cohorts were derived from a special group or there was no description of how the interval was calculated. This domain also evaluates the certitude that the cohorts did not have the outcome at the beginning of the study. However for our review all studies were at low risk of bias since the outcome is pre-eclampsia in the second pregnancy and women were included in the study on the basis of pre-eclampsia in the first pregnancy.

The second domain assesses comparability of the cohorts. We defined a study as at low risk of bias if the study controlled for at least variables affecting pre-eclampsia such as age or socioeconomic status and at high risk of bias if results were not adjusted.

The third domain relates to the outcome and it evaluates three aspects, outcome definition, time to develop the outcome and adequate follow up. We classified the study as at low risk of bias if there was a clear definition of pre-eclampsia and eclampsia, or if authors reported assessment on the basis of the ICD-10 codes. For the second aspect we considered a study at low risk of bias if the study allowed enough time for women to have a second pregnancy and if the study allowed measurement of pre-eclampsia in the postpartum period. Studies were also at low risk of bias if they had less than 20 % lost to follow up or if the study reported that the subjects lost to follow up were unlikely to introduce bias.

### Strategy for analysis and data synthesis

The association between inter-pregnancy interval and recurrent pre-eclampsia was calculated. Meta-analysis of adjusted and unadjusted odds ratios (aOR or OR) with 95 % confidence intervals (CI) calculated using the generic inverse variant method, was used to explore the association between various interval lengths and adverse outcome [24]. We did not plan a reference inter-pregnancy interval a priori; instead we used the intervals considered in the original studies. If inter-pregnancy intervals were defined differently in the articles we planned to perform a meta-regression analysis considering the exposure time [25]. If the same data set was presented in multiple reports, that providing the most information was considered. Where the data available did not allow us to perform a meta-analysis (e.g. incompatible birth intervals reported), we presented data without further attempt to quantitatively synthesize it.

Sub-group analysis by stillbirth and change of partner were performed. Interaction of other factors were assessed using multivariate analysis.

## Results

**Fig. 1:**
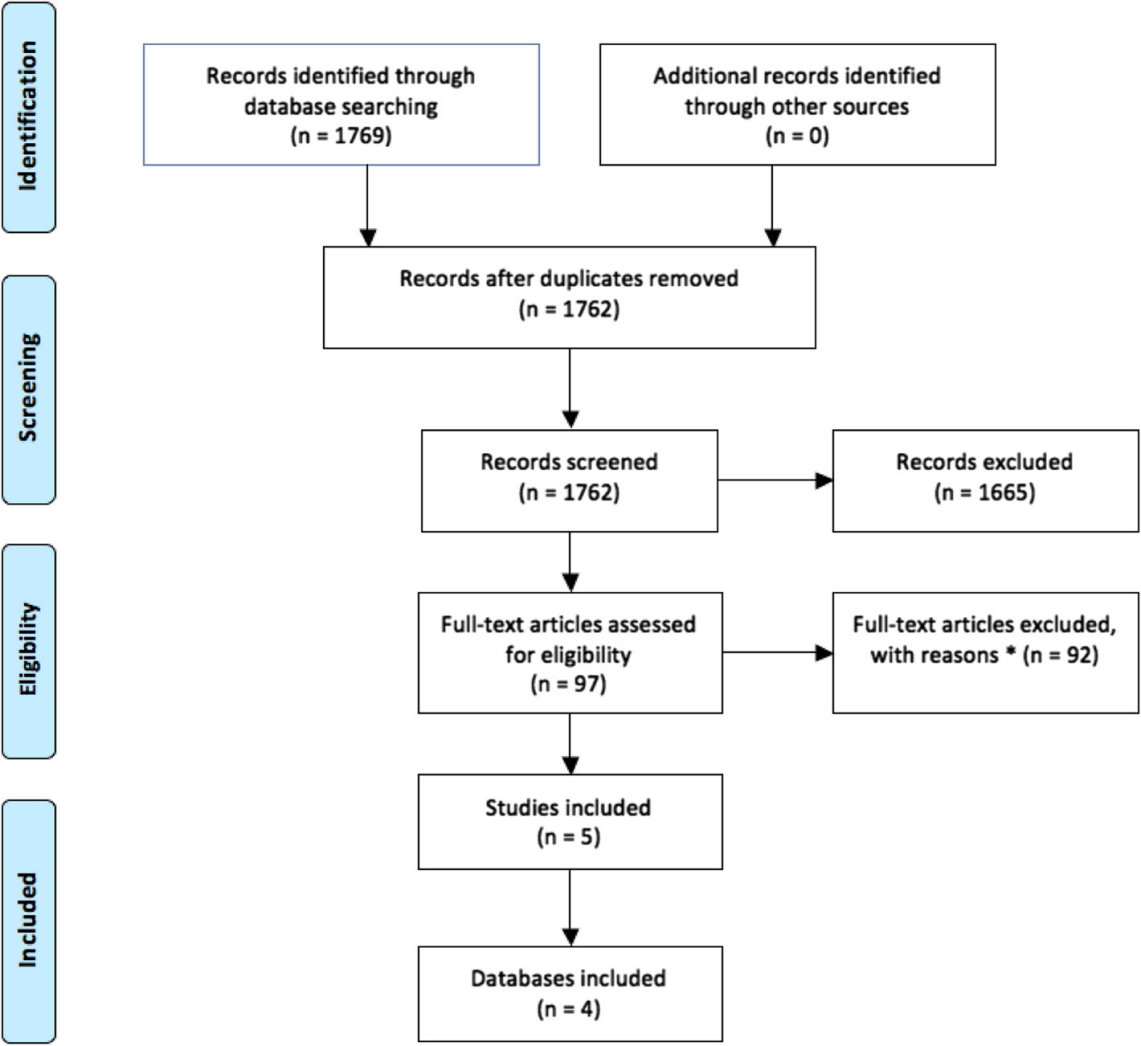
Study selection process for systematic review of the inter-pregnancy interval and risk of recurrent pre-eclampsia. PRISMA 2009 Flow Diagram

The search strategy retrieved a total of 1769 articles from which 97 were selected for full text evaluation and finally five articles [12–14, 26, 27] from four datasets were included in the review (Fig. 1). Two of the five articles reported results from the same dataset [13, 26]; however, only the article reporting adjusted results was included [26]. The main characteristics of the included studies are shown in Table 1.

**Table 1:** Main characteristics of included studies

Study	Country	Study design	Sample size (women with PE in 1st pregnancy)	Definition of inter-pregnancy interval	Inter-pregnancy interval studied (years)	Elegibility criteria in pregnancy 1	Outcome in pregnancy 2	Definition of outcome	Confounders used in the adjustment
Basso 2001	Denmark	Retrospective cohort	8401	Interval between birth of the first child and conception date of the second one	≤1, 1–2, 2-3, 3–4, 4-5, 5–7, > 7.	Danish women with PE that delivered at the hospital, with a 28 weeks or more single pregnancy and whose children were not given in adoption who had subsequently given birth	PE or E	ICD-9 and ICD-10	Maternal age in the second pregnancy, SES in the first pregnancy and change of SES between the first and second pregnancies.
Mostello	USA	Retrospective cohort	17,773	Interval between birth of the first child and conception date of the second one	≤1, 1–2, 2-3, 3–4, 4-5, 5–6, 6-7, 7–8, > 8.	Women who delivered their first 2 singleton pregnancies at > 20 weeks gestation	PE or E	PE = HT + proteinuria or edema that is generalized and overt	maternal age, prepregnancy BMI, smoking and SES
								E = PE + convulsion, coma or both	
Trogstad	Norway	Retrospective cohort	19,970	Time between the dates of the two deliveries	≤1, 1–5, 6-10, 11-15, > 15	All women with a first and second singleton pregnancy delivery after 16 weeks gestation in Norway	PE or E	BP > 140/90 after 20 week gestation combined with proteinuria ≥0.3 g/24 h (≥ + 1 dipstick) on at least two occasions. ICD-8	maternal age in 2nd pregnancy, new father, year of second delivery.
Hernandez Diaz	Sweden	Retrospective cohort	31,417	Difference between dates of last menstrual period	<2, 2-4, 4-6, 6-8, > 8	Women who had their first delivery on or after 1 January 1987, with reproductive history followed until the end of 2004.	PE or E	ICD-9 and ICD-10	None

References: *PE* pre-eclampsia, *HT* hypertension, *E* Eclampsia, *ICD* International Classification of Disease, *SES* Socioeconomic status

All studies were from high income countries, namely Norway [12], Denmark [14], Sweden [26] and United States [27]. All were retrospective cohorts of women with a singleton pregnancy diagnosed with pre-eclampsia or eclampsia and a subsequent singleton pregnancy. No other exclusion criteria in terms of other hypertension disorders of pregnancy (e.g. pre-existing hypertension) was mentioned in the original articles. Sample sizes ranged from 8401 to 31,417. Two studies reported data separately for pregnancies with the same partner or different partner [12, 14]. Two studies [14, 26] defined inter-pregnancy interval as the time between the birth of the first child and conception of the second one; one study [12] defined the interval as the time between deliveries calculated by subtracting the dates of both births; and a further study [27] used the difference between the dates of the last menstrual periods of both pregnancies.

**Fig. 2:**
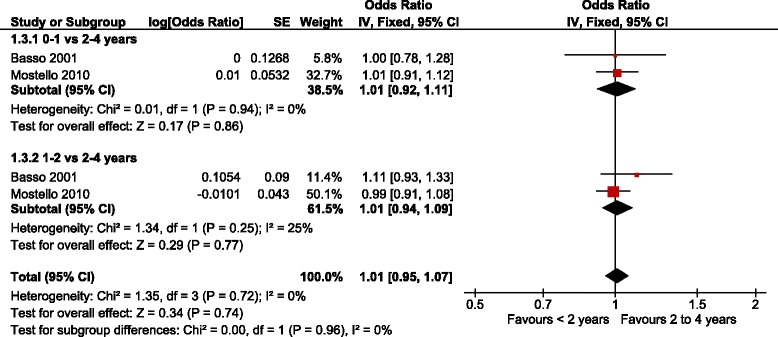
Adjusted odds ratio of recurrent pre-eclampsia with inter-pregnancy interval below 2 years compared to 2–4 years (i.e. 0–1 and 1–2 years)

**Fig. 3:**
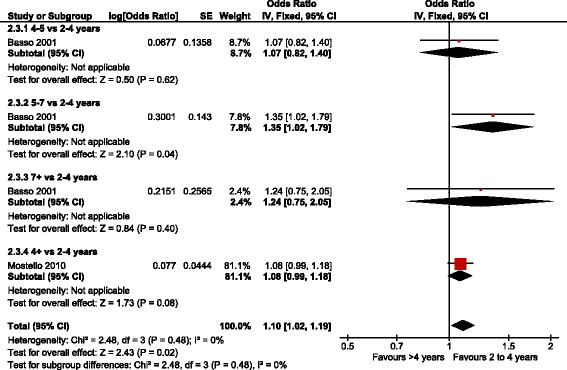
Adjusted odds ratio of recurrent pre-eclampsia with inter-pregnancy intervals greater than 4 years compared to 2–4 years

Two of the four datasets [14, 26] were included in the meta-analysis with a total of 26,174 singleton pregnancies. Basso et al. included a cohort of Danish women with a singleton pregnancy of gestational age of 27 completed weeks or above, diagnosed with pre-eclampsia or eclampsia and a subsequent pregnancy with information on the partner in both births between 1980 and 1994. However the OR for recurrent pre-eclampsia was reported only for those women that did not change their partner. The results were adjusted by maternal age and socioeconomic status in the first pregnancy and change of socioeconomic status between both pregnancies. Mostello et al. included a retrospective cohort with consecutive singleton pregnancies of more than 20 weeks of gestation diagnosed with pre-eclampsia or eclampsia and a subsequent pregnancy between January 1989 and December 2005. Results were adjusted by maternal age, prepregnancy Body Mass Index (BMI), weight gain, smoking, chronic disease, and socioeconomic status. Four percent of this cohort had chronic hypertension. Figures 2 and 3 present the forest plots of these two studies with different comparisons. The aOR for recurrent pre-eclampsia with an inter-pregnancy interval of less than 2 years compared to 2-4 years was 1.01 [95 % CI 0.95 to 1.07] (*N* = 26,174) with low heterogeneity (*P* = 0.72; *I*^2^ = 0 %). No differences were found when the interval of less than 2 years was subdivided in two subgroups (less than 1 year or 1 to 2 years) and compared to the 2-4 years interval (Fig. 2). The test for subgroup differences shows a p value of 0.96 (*I*^2^ = 0 %) implying a similar effect for subgroups. The aOR of recurrent pre-eclampsia with an inter-pregnancy interval of more than 4 years compared to 2–4 years was 1.10 [95 % CI 1.02 to 1.19], with 26,174 women and low heterogeneity (*P* = 0.48; *I*^2^ = 0 %) (Fig. 3).

Two of the four datasets [12, 27] with a total of 51,387 singleton pregnancies were not included in the meta-analysis. Trogstad et al. included a retrospective cohort of pregnancies of more than 16 weeks of gestation with diagnosis of pre-eclampsia or eclampsia in the first pregnancy and a subsequent singleton pregnancy between 1967 and 1998. This study was not included in the meta-analysis as the reference interval used to calculate the OR was very different from the other included studies (1 to 5 years) [12]. The results were adjusted by maternal age, same father and year of the second delivery and they are reported in Table 2. The aOR of recurrent pre-eclampsia for an inter-pregnancy interval of less than 1 year compared to 1 to 5 years was 1.24 [95 % CI 0.83 to 1.85] (*N* = 19,970). When longer intervals were compared with intervals of 1 to 5 years, the aOR was 1.04 [95 % CI 0.93 to 1.16] for intervals of 6 to 10 years; 0.85 [95 % CI 0.61 to 1.19] for intervals of 11 to 15 years and 0.78 [95 % CI 0.48 to 1.27] for intervals longer than 15 years.

**Table 2:** Trostad 2001. Odds ratio of recurrent pre-eclampsia with inter-pregnancy intervals of less than 1 or 5 years or more compared to 1–5 years. Results were adjusted by maternal age in 2nd pregnancy, new father, delivery year

Interval	OR	Low	High	Sub-group	OR	Low	High	Sub-group	OR	Low	High
≤1 year	1.24	0.83	1.85	Same father	1.18	0.76	1.85	New father	-	-	-
1–5 years	Ref.	Ref.	Ref.		Ref.	Ref.	Ref.		Ref.	Ref.	Ref.
6–10 years	1.04	0.93	1.16		1.06	0.93	1.21		1.02	0.65	1.61
11–15 years	0.85	0.61	1.19		0.52	0.27	1.01		0.63	0.29	1.4
>15 years	0.78	0.48	1.27		0.73	0.28	1.91		0.52	0.19	1.41

Hernandez-Díaz et al. included a cohort of pregnancies from the first antenatal visit (usually at 8 to 12 weeks’gestation) with diagnosis of pre-eclampsia or eclampsia and a subsequent pregnancy between January 1987 and December 2004 [27]. The study reports a 14.7 % risk of recurrent pre-eclampsia. For those women with a history of pre-eclampsia in the first pregnancy the risk of recurrence was 13.1 % if they became pregnant within 2 years and 15.8 % if the next pregnancy was after 8 years or later (Table 3). The authors did not report ORs or the total number of women in each group, which would have allowed their calculation. We contacted the authors but they no longer had access to the database to perform the analysis required for inclusion in the meta-analysis.

**Table 3:** Hernández Díaz. Risk of recurrent pre-eclampsia in second pregnancy by years since first pregnancy

Interval	%	Low	High
< 2 years	13.13	12.26	14.00
2 to 4 years	14.87	14.20	15.54
4 to 6 years	16.62	15.01	18.23
6 to 8 years	15.96	13.18	18.75
> 8 years	15.85	12.39	19.32

None of the studies reported the incidence of hypertension or perinatal outcomes in the second pregnancy by inter-pregnancy intervals.

### Change of partner and risk of pre-eclampsia or eclampsia

Two studies assessed the risk of recurrent pre-eclampsia according to inter-pregnancy interval and change in partner. Togstad et al. found similar results when the analysis was performed separately for those pregnancies with the same partner or when the partner changed between the first and the second pregnancy (Table 2). Baso et al. in 2001 also reported very little effect of changing partner on the risk of pre-eclampsia, although it seemed to be protective in those intervals longer than 5 years OR 0.56 (0.37-0.85). The OR of recurrent pre-eclampsia associated with changing partner was 0.80 (CI 95 % 0.63-1.00) after adjustment for inter-pregnancy interval.

### Risk of bias assessment

**Fig. 4:**
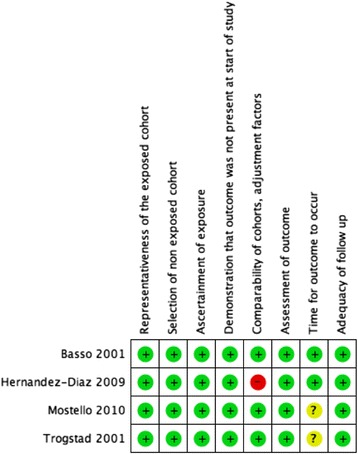
Methodological quality of included studies according to Newcastle-Ottawa Scale criteria

**Fig. 5:**
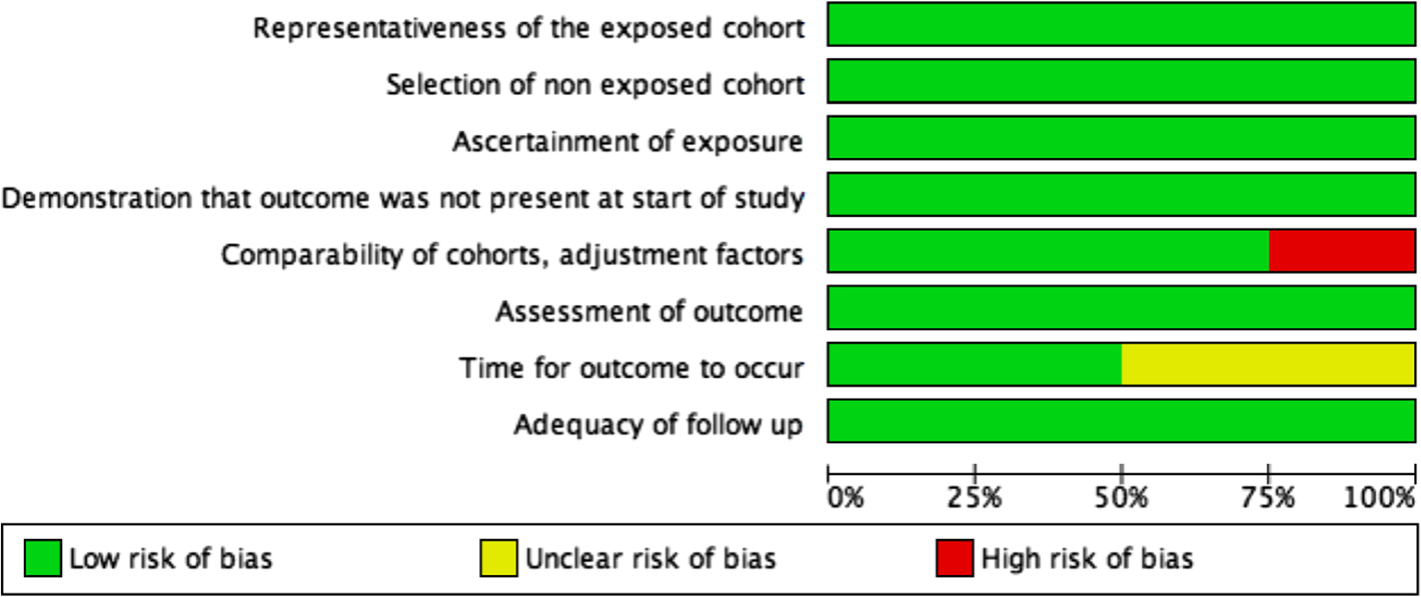
Summary of methodological quality assessment of risk of bias and applicability concerns presented for each domain as percentages across all included studies

Quality assessment of included studies are described in Figs. 4 and 5. For the first domain, all studies were classified as at low risk of bias as all were large retrospective cohorts derived from the general community with the non-exposed cohort belonging to the same group as the exposed cohort. All of the studies included a description of how the inter-birth or inter-pregnancy interval was calculated and how dates were extracted from clinical records.

Three studies [12–14] were classified as at low risk of bias as results were adjusted by at least age of the mother and socioeconomic status and one study [27] was at high risk of bias as results were not adjusted.

All studies had a clear definition of pre-eclampsia or eclampsia and three studies used the international classification of disease to define the outcome. They all allowed enough time for women to have a second pregnancy as the cohort periods were between 14 and 30 years long. Basso et al. and Hernandez Diaz et al. assessed pre-eclampsia or eclampsia at discharge and were classified as at low risk of bias whereas Trogstad et al. evaluated pre-eclampsia or eclampsia “at delivery ward within 1 week of delivery” and Mostello et al. did not specify the timing of evaluation, and were classified as at unknown risk. All studies were also considered at low risk of bias due to loss to follow up as the loss to follow up was unlikely to introduce bias.

## Discussion

### Main Findings

This review identified five reports using four databases which assessed the incidence of recurrent pre-eclampsia according to inter-pregnancy interval. All studies reported data from retrospective cohorts in high income countries. Only two of the four datasets could be meta-analysed concluding that when comparing inter-pregnancy interval below 2 years with intervals of 2–4 years, there is no significant increase in risk (aOR = 1.01 [95 % CI 0.95 to 1.07]). On the other hand, when comparing intervals of more than 4 years with intervals of 2–4 years, we found a significant small increase, aOR = 1.10 [95 % CI 1.02 to 1.19]. We found no heterogeneity between these estimates.

Change in partner between pregnancies has been suggested to confound the relationship between inter-pregnancy interval and pre-eclampsia, as change of partner is often associated with longer interpregnancy intervals [28, 29]. Since the incidence of pre-eclampsia is much higher in first pregnancies, it has been suggested that the decrease in risk in the second pregnancy is found only if the father remains the same, the hypothesis being that the maternal immune system builds tolerance to the paternal antigens with repetitive exposure which is not the case if the partner changes.

However the relationship may be more complex, in that one needs to consider both the inherent propensity to pre-eclampsia of each specific couple, and the modifying effect of prolonged immunological exposure. The above argument would hold when considering an unselected population. In the case of a cohort selected on the basis of previous pre-eclampsia, we would suggest that the primary partnerships are selected as above average risk. In this case, a change in partner may result in a partnership with less inherent propensity to pre-eclampsia (on the principle of regression to the mean). This may outweigh the effect of less immunological exposure within the new partnership, resulting in lower rather than increased risk of recurrence. Therefore a change of partner, with a sufficient period of vaginal exposure to seminal antigens may decrease the likelihood of pre-eclampsia [30].

However, despite the hypothesis above, it has also been argued that change in paternity is not a confounder but a collider and thus controlling for this variable could be inappropriate and produce spurious results [31]. In our review, we found two studies which assessed the relationship between inter-pregnancy interval and recurrent pre-eclampsia controlling for change of partner but we did not find a statistical nor clinical significant difference [12, 14].

### Strengths and limitations

Strengths of this review include the broad search strategy to capture the largest number of publications and the relatively good quality of the original observational studies: large retrospective population-based cohorts within a period of 15 to 30 years. We tried to reduce bias by screening and extracting the information in duplicate using a data-extraction form specifically designed for this review. Additionally, the meta-analysis included studies with proper adjustment for confounding variables.

This review has several limitations. Only four datasets studies were retrieved and all were from high income countries. Exposure was defined differently in the included studies which restricted the analysis and interpretation. Only two of the four datasets were included in the meta-analysis as inter-pregnancy intervals were defined differently in one study and data was not adjusted in another study. We planned to perform a meta-regression analysis considering the exposure time, however we did not have a sufficient number of studies for this type of analysis. Furthermore, reported aOR were not adjusted for the same confounders across the studies, nor did they include all confounding factors known to play a role in the association between inter-pregnancy intervals and pre-eclampsia or eclampsia. Three datasets were adjusted for maternal age, one adjusted also for change of partner and delivery year [12], a second one for socioeconomic status, BMI and smoking [26] and a third one for socioeconomic status [14].

In addition, other methodological limitations remain. In the case of inter-birth intervals, it is important to consider the confounding effect of complications in the subsequent pregnancy leading to early delivery, which creates bias towards a shorter inter-pregnancy interval. For future research, we recommend that interpregnancy interval (birth to commencement of the next pregnancy) be used, as this interval is most useful to parents planning another pregnancy.

Also, the “short inter-pregnancy intervals” may have been too broad and there was not enough data looking at intervals of less than 1 year. Inter-pregnancy intervals of less than 1 year would not be captured with the data available for this meta-analysis. This is important since some evidence has suggested that a period of 6 months of unprotected sexual cohabitation may be enough to decrease the risk of pre-eclampsia [32].

The analysis and interpretation of the data gathered by this review would have been enhanced by including information on chronic or pre-existing hypertension, onset of pre-eclampsia (early vs. late) as well as outcomes such as stillbirth but unfortunately, there was not enough information to explore subgroup analysis using these variables [33].

### Interpretation (in light of other evidence)

This is the first systematic review that assess the risk of recurrent pre-eclampsia/eclampsia according to inter-pregnancy or birth intervals. These results are in accordance with those of a large cross-sectional study in Latin America which assessed the relationship between inter-pregnancy interval and ocurrence of pre-eclampsia and other various maternal and perinatal outcomes. Without considering the outcome of the previous pregnancy, the above-mentioned study reported increased odds of pre-eclampsia associated with longer inter-pregnancy intervals [8]. Bearing in mind the scarcity of studies and the limitations of the data available, the updated analysis information provided in this systematic review is important as it has been reported that intervals shorter than 2 years or longer than 10 years increased the risk of recurrent pre-eclampsia [16, 34]. In addition, even though among the risk factors for recurrent pre-eclampsia, inter-pregnancy interval may be regarded as a minor contributor, it is nonetheless, together with weight control, a modifiable factor through which to intervene before conception.

## Conclusion

Data from large population-based cohorts show evidence that when compared to inter-pregnancy intervals of 2 to 4 years, shorter intervals are not associated with an increased risk of recurrent pre-eclampsia. However, the risk appears to increase in longer inter-pregnancy intervals. The results of this review should be interpreted with caution as the data available is limited and derives from observational studies and thus subject to possible confounding factors. Future research is requiered to confirm our findings and to explore inter-pregnancy intervals shorter than 1 year.

## Additional file

Additional file 1: Search Strategy. (PDF 110 kb)
