# Supplementary material for: Inter-pregnancy interval and risk of recurrent pre-eclampsia: systematic review and meta-analysis
Source: Reprod Health. 2016 Jul 18;13:83. doi: 10.1186/s12978-016-0197-x (PMC4950816; doi:10.1186/s12978-016-0197-x)
Supplement: Additional file 1: — Search Strategy. (PDF 110 kb) [file 12978_2016_197_MOESM1_ESM.pdf]

Estrategias Eclampsia

PubMed

Search	Query	Items found
#25	Search **(#12 AND #24)**	611
#24	Search **(#13 OR #14 OR #15 OR #16 OR #17 OR #18 OR #19 OR #20 OR #21 OR #22 OR #23)**	95629
#23	Search **Interpregnancy Space*[tiab]**	7
#22	Search **Interpregnancy Interval*[tiab]**	228
#21	Search **Timing[tiab]**	83821
#20	Search **Subsequent Pregnan*[tiab]**	3263
#19	Search **Reproductive Pattern*[tiab]**	413
#18	Search **Intergenesic Interval*[tiab]**	12
#17	Search **Delivery Spac*[tiab]**	5551
#16	Search **Delivery Interval*[tiab]**	810
#15	Search **Birth Spac*[tiab]**	595
#14	Search **Birth Interval*[tiab]**	1007
#13	Search **Birth Intervals[Mesh]**	1344
#12	Search **(#1 OR #2 OR #3 OR #4 OR #5 OR #6 OR #7 OR #8 OR #9 OR #10 OR #11)**	39116
#11	Search **Preeclampsia*[tiab]**	12543
#10	Search **Gestosis[tiab]**	1202
#9	Search **Pregnancy Toxemia*[tiab]**	1239
#8	Search **EPH[tiab]**	2205
#7	Search **Pre Eclampsia*[tiab]**	7197
#6	Search **Eclampsia*[tiab]**	11032
#5	Search **Pregnancy-Induced Hypertension[tiab]**	3018
#4	Search **Maternal Hypertens*[tiab]**	518
#3	Search **Gestational Hypertension[tiab]**	1435
#2	Search **Transient Hypertension[tiab]**	366
#1	Search **Hypertension, Pregnancy-Induced[Mesh]**	27494

Embase

No. Query Results Results Date

#25. 12 AND 24 508

#24. OR/13-23	133,582
#23. 'interpregnancy space$':ab,ti	0
#22. 'interpregnancy interval$':ab,ti	239
#21. 'timing':ab,ti	99,821
#20. 'subsequent pregnan$':ab,ti	0
#19. 'reproductive pattern$':ab,ti	138
#18. 'intergenesic interval$':ab,ti	11
#17. 'delivery spac$':ab,ti	0
#16. 'delivery interval$':ab,ti	945
#15. 'birth spac$':ab,ti	0
#14. 'birth interval$':ab,ti	528
#13. 'family planning'/exp	32,580
#12. OR/1-11	41,532
#11. 'pregnancy toxemia$':ab,ti	628
#10. 'gestosis':ab,ti	1,456
#9. preeclampsia$:ab,ti	18,645
#8. eph:ab,ti	2,641
#7. 'pre eclampsia$':ab,ti	9,393
#6. 'eclampsia$':ab,ti	14,001
#5. 'pregnancy-induced hypertension$':ab,ti	3,788
#4. 'maternal hypertens$':ab,ti	0
#3. 'gestational hypertension':ab,ti	2,114
#2. 'transient hypertension':ab,ti	416
#1. 'maternal hypertension'/exp	10,173

Cochrane

ID	Search	Hits
#1	MeSH descriptor: [Hypertension, Pregnancy-Induced] explode all trees	713
#2	Transient Hypertension:ti,ab,kw (Word variations have been searched)	542
#3	Gestational Hypertension:ti,ab,kw (Word variations have been searched)	365
#4	Maternal Hypertens*:ti,ab,kw (Word variations have been searched)	412
#5	Pregnancy-Induced Hypertension:ti,ab,kw (Word variations have been searched)	318
#6	Eclampsia*:ti,ab,kw (Word variations have been searched)	918
#7	Pre Eclampsia*:ti,ab,kw (Word variations have been searched)	815
#8	EPH:ti,ab,kw (Word variations have been searched)	21
#9	Pregnancy Toxemia*:ti,ab,kw (Word variations have been searched)	31
#10	Gestosis:ti,ab,kw (Word variations have been searched)	24
#11	Preeclampsia*:ti,ab,kw (Word variations have been searched)	638
#12	#1 or #2 or #3 or #4 or #5 or #6 or #7 or #8 or #9 or #10 or #11	2284
#13	MeSH descriptor: [Birth Intervals] explode all trees	12
#14	Birth Interval*:ti,ab,kw (Word variations have been searched)	1635
#15	Birth Spac*:ti,ab,kw (Word variations have been searched)	68
#16	Delivery Interval*:ti,ab,kw (Word variations have been searched)	2468
#17	Delivery Spac*:ti,ab,kw (Word variations have been searched)	336
#18	Intergenesic Interval*:ti,ab,kw (Word variations have been searched)	0
#19	Reproductive Pattern*:ti,ab,kw (Word variations have been searched)	76
#20	Subsequent Pregnan*:ti,ab,kw (Word variations have been searched)	804
#21	Timing:ti,ab,kw (Word variations have been searched)	189095
#22	Interpregnancy Interval*:ti,ab,kw (Word variations have been searched)	3
#23	Interpregnancy Spac*:ti,ab,kw (Word variations have been searched)	0
#24	#13 or #14 or #15 or #16 or #17 or #18 or #19 or #20 or #21 or #22 or #23	191973

#25	#12 and #24	607

LILACS

Base de datos :	**LILACS**
Búsqueda :	**(MH Hipertensión Inducida en el Embarazo OR Transient Hypertension OR Hipertensión Gestacional OR Hipertensão Gestacional OR Gestational Hypertension OR Pregnancy-Induced Hypertension OR Gestosis OR Eclampsia$ OR Pre Eclampsia$ OR Pregnancy Toxemia$ OR Preeclampsia$) AND (MH Intervalo entre Nacimientos OR Birth Spac$ OR Birth Interval$ OR Delivery Interval$ OR Delivery Spac$ OR Intergenesic Interval$ OR Reproductive Pattern$ OR Subsequent Pregnan$ OR Espaçamento entre Nascimentos OR Padrão Reprodutivo OR Gravidez Subseqüente OR Patrón Reproductivo OR Timing OR Intervalo Intergenésico OR Interpregnancy Spac$ OR Interpregnancy Interval$) [Palabras]**
Referencias encontradas :	**2** [refinar]
